# Artificial intelligence in future nursing care: Exploring perspectives of nursing professionals - A descriptive qualitative study

**DOI:** 10.1016/j.heliyon.2024.e25718

**Published:** 2024-02-08

**Authors:** Moustaq Karim Khan Rony, Ibne Kayesh, Shuvashish Das Bala, Fazila Akter, Mst. Rina Parvin

**Affiliations:** a Master of Public Health, Bangladesh Open University, Gazipur, Bangladesh; b Institute of Social Welfare and Research, University of Dhaka, Dhaka, Bangladesh; c Associate Professor, International University of Business Agriculture and Technology, Dhaka, Bangladesh; d Dhaka Nursing College, affiliated with the University of Dhaka, Bangladesh; e Afns Major at Bangladesh Army, Combined Military Hospital, Dhaka, Bangladesh; f School of Medical Sciences, Shahjalal University of Science and Technology, Sylhet, Bangladesh

**Keywords:** Artificial intelligence, Perspectives, Nursing professionals, Patient care

## Abstract

**Background:**

The healthcare landscape is rapidly evolving, with artificial intelligence (AI) emerging as a transformative force. In this context, understanding the viewpoints of nursing professionals regarding the integration of AI in future nursing care is crucial.

**Aims:**

This study aimed to provide insights into the perceptions of nursing professionals regarding the role of AI in shaping the future of healthcare.

**Methods:**

A cohort of 23 nursing professionals was recruited between April 7, 2023, and May 4, 2023, for this study. Employing a thematic analysis approach, qualitative data from interviews with nursing professionals were analyzed. Verbatim transcripts underwent rigorous coding, and these codes were organized into themes through constant comparative analysis. The themes were refined and developed through the grouping of related codes, ensuring an authentic representation of participants' viewpoints.

**Results:**

After careful data analysis, ten key themes emerged including: (I) Perceptions of AI readiness; (II) Benefits and concerns; (III) Enhanced patient outcomes; (IV) Collaboration and workflow; (V) Human-tech balance: (VI) Training and skill development; (VII) Ethical and legal considerations; (VIII) AI implementation barriers; (IX) Patient-nurse relationships; (X) Future vision and adaptation.

**Conclusion:**

This study provides valuable insights into nursing professionals' perspectives on the integration of AI in future nursing care. It highlights their enthusiasm for AI's potential benefits while emphasizing the importance of ethical and compassionate nursing practice. The findings underscore the need for comprehensive training programs to equip nursing professionals with the skills necessary for successful AI integration. Ultimately, this research contributes to the ongoing discourse on the role of AI in nursing, paving the way for a future where innovative technologies complement and enhance the delivery of patient-centered care.

## Highlights

• Nurses envisioned AI's potential for early detection, saving lives, and relieving routine tasks, but expressed concerns about overlooking nuances and preserving holistic care.
• Nurses recognized the imperative of training for AI integration, actively seeking opportunities to enhance understanding, showcasing a proactive approach to embracing technological advancements.
• Nurses undertook a comprehensive exploration of ethical and legal dimensions, advocating for patient privacy, data security, and a responsible, ethical use of AI in nursing.
• Nurses recognized obstacles like limited resources and resistance to change in AI integration, emphasizing a collaborative and proactive approach to overcome implementation challenges.
• Nurses advocated for a harmonious AI integration that enhances patient interactions without substituting compassionate care, emphasizing the irreplaceable human touch in nursing practice.

## 1 Introduction

Artificial Intelligence (AI) has seen a meteoric rise in popularity in recent years, transforming industries across the board. Its potential to revolutionize healthcare, a field intrinsically linked with human well-being, is especially promising [1]. Nursing, at the frontline of patient care, stands to benefit considerably from the integration of AI technologies [2,3]. AI has catapulted into the spotlight due to its multifaceted capabilities. AI involves the development of computer systems that can emulate human intelligence, encompassing tasks such as problem-solving, pattern recognition, and decision-making [4,5]. The surge in AI's popularity can be attributed to remarkable advancements in machine learning, deep learning, and natural language processing, making it a transformative force in fields as diverse as finance, manufacturing, entertainment, and, crucially, healthcare [6,7].

In the context of nursing, AI represents an amalgamation of cutting-edge technologies, promising to redefine the way healthcare is delivered [8]. AI in nursing entails the utilization of AI-powered tools, algorithms, and systems to assist nurses in their multifarious roles, spanning clinical care and administrative duties [9]. These AI applications encompass a wide spectrum, including but not limited to electronic health record (EHR) management, medication administration [10] and predictive analytics such as predicting falls and pressure injuries [11]. One of the primary motivations driving the integration of AI into nursing is its potential to elevate the quality of patient care [12]. AI systems are adept at processing vast volumes of patient data swiftly, which enables timely and accurate clinical decision support. For instance, AI algorithms can sift through patient records, identify trends, predict disease progression, and suggest personalized treatment plans [13]. This not only alleviates the cognitive load on nurses but also heightens the precision and efficiency of care delivery [14].

Moreover, AI-powered devices and sensors enable continuous patient monitoring, offering real-time updates on vital signs and instant alerts to healthcare providers in cases of abnormalities [15]. This proactive approach to patient monitoring can lead to early interventions, ultimately translating into improved patient outcomes [16]. Additionally, AI-driven chatbots and virtual assistants can engage with patients, responding to queries and providing educational resources, thereby enhancing patient engagement and health literacy [17]. The infusion of AI into nursing isn't solely aimed at optimizing healthcare processes; it also has a profound impact on fostering positive patient outcomes [18]. AI plays a pivotal role in preventive care, ensuring patients receive timely screenings and interventions. Through predictive modeling, AI can identify individuals at risk of chronic conditions or readmissions, allowing nurses to tailor interventions and educational outreach accordingly [19]. Furthermore, AI facilitates nurses in managing their workloads more efficiently. Automation of routine tasks, such as medication reminders and documentation, allows nurses to dedicate more time to direct patient care, thereby promoting a more patient-centered approach [20,21]. This shift in focus is instrumental in improving patient satisfaction and enhancing the overall healthcare experience [22].

The rationale underpinning this study is grounded in recognizing a critical juncture where technology, specifically AI, intersects with the essential realm of nursing care. As AI's popularity continues to soar across various sectors, including healthcare, understanding its impact on nursing practice and patient care becomes paramount [23]. Nursing, as the backbone of healthcare, finds itself uniquely positioned to harness AI's potential for optimizing patient outcomes. However, despite the evident enthusiasm for AI in healthcare, a gap exists in qualitative research focusing on the perspectives of nursing professionals. This study aimed to address this void by delving into the nuanced and diverse viewpoints of nursing professionals concerning AI's integration into their practice. Nurses bring rich and varied experiences from different specialties, making their perspectives invaluable in shaping the future of nursing care in the era of AI. Through the exploration of their perspectives, this study will contribute to a more comprehensive understanding of AI's role in nursing and lay a valuable foundation for future initiatives.

## 2 Methodology

### 2.1 Research design

In this study, a descriptive qualitative approach was employed, which included thematic analysis. The researchers ensured that the Consolidated Criteria for Reporting Qualitative Research (COREQ) checklist was followed throughout the study to enhance the rigor and transparency of the qualitative research process. Qualitative research, known for its suitability in delving into intricate and nuanced phenomena [24], was particularly chosen to investigate the perspectives of nursing professionals concerning the integration of AI in future nursing care. Thematic analysis was chosen to systematically extract and analyze recurring themes within the qualitative data [25]. In this study, thematic analysis allowed researchers to uncover and comprehend the complex viewpoints and insights held by nursing professionals concerning the role of AI in future nursing care, facilitating a comprehensive understanding of their perspectives.

The study was conducted at three tertiary-level private hospitals in Dhaka city, Bangladesh. The initial step involved compiling a roster containing nurses' email addresses and phone numbers, a task accomplished by liaising with the hospital administration. This compilation served as the foundation for participant recruitment, allowing for the engagement of nurses in face-to-face interviews. By gathering this contact information, the study established a direct channel to engage potential participants and gather their perspectives regarding AI's integration in nursing care within the specific healthcare context.

### 2.2 Sampling strategy

A purposive sampling strategy was employed to select participants for this study. This method involved the intentional selection of individuals with pertinent knowledge and experience in the domains of nursing care and AI. Through this approach, the sample was curated to encompass individuals capable of offering substantial and valuable insights into the research topic [26]. The utilization of purposive sampling ensured that the study engaged participants who could contribute comprehensively to the exploration of the integration of AI in nursing care.

### 2.3 Sample size

In this study, a participant cohort of 23 nursing professionals was recruited from April 7, 2023, to May 4, 2023. This specific sample size had been deemed sufficient to achieve data saturation, a point at which further interviews ceased to yield novel significant insights or themes, and to facilitate a comprehensive understanding of the research phenomenon [27]. The selected sample size was strategically determined to enable a thorough exploration of nursing professionals' perspectives, offering a rich and multifaceted insight into their viewpoints concerning the integration of AI in future nursing care. By accommodating a diverse range of sociodemographic information including experiences, educational backgrounds within this size (Table 1), this study aimed to provide a holistic understanding of the subject matter. Furthermore, the careful selection of this sample size allowed for a balanced approach, taking into account the depth of insights alongside practical considerations like time constraints and resource availability [28]. This approach was grounded in the aspiration to derive valuable and meaningful insights while ensuring the research process's feasibility and manageability [29].

**Table 1:** Provides an overview of the participants' demographics, professional backgrounds, and major findings or perspectives regarding the integration of AI in nursing care.

Participants	Age	Sex	Educational background	Designation	Working experience (years)	Working department	Major finding/Perspectives
Participant 1	34	Female	Masters in Public Health	Senior staff nurse	8	Surgical Unit	Perceptions of AI readiness, Collaboration and workflow
Participant 2	42	Male	Master of Science in Nursing	Nurse Manager	15	Intensive care unit	Training and skill development
Participant 3	28	Female	Masters in Public Health	Senior staff nurse	5	Pediatrics	Future vision and adaptation, Ethical and legal considerations
Participant 4	56	Female	Doctor of Nursing Practice	Lecturer	30	Education	Perceptions of AI readiness, Human-tech balance, AI implementation barriers
Participant 5	39	Male	Masters in Public Health	Charge nurse	12	Emergency Department	Patient-nurse relationships, Collaboration and workflow
Participant 6	47	Female	Masters in Nutrition	Nurse manager	20	Coronary care unit	Enhanced patient outcomes, Future vision and adaptation
Participant 7	31	Female	Masters in Public Health	Senior staff nurse	6	Oncology	Enhanced patient outcomes
Participant 8	33	Male	Masters in Public Health	Senior staff nurse	7	Coronary care unit	Training and skill development, AI implementation barriers
Participant 9	36.5	Female	Doctor of Nursing Practice	Lecturer	14	Education	Benefits and concerns, Enhanced patient outcomes, Ethical and legal considerations
Participant 10	40	Female	Master of Science in Nursing	Clinical instructor	14	Education	Patient-nurse relationships, Enhanced patient outcomes
Participant 11	28.5	Male	Masters in Nutrition	Charge nurse	5.5	Neurology	Perceptions of AI readiness, AI implementation barriers
Participant 12	50	Female	Master of Science in Nursing	Nurse manager	22	Obstetrics	Ethical and legal considerations, Training and skill development
Participant 13	37	Male	Doctor of Nursing Practice	Lecturer	10	Education	Training and skill development, Patient-nurse relationships
Participant 14	35	Female	Master of Science in Nursing	Senior staff nurse	9	Orthopedics	Benefits and concerns
Participant 15	45	Female	Masters in Public Health	Charge nurse	18	Pediatrics	Collaboration and workflow, Future vision and adaptation
Participant 16	27	Male	Masters in Public Health	Lecturer	8	Education	Ethical and legal considerations, Training and skill development
Participant 17	32	Female	Masters in Public Health	Senior staff nurse	5	ICU	Human-tech balance, AI implementation barriers
Participant 18	38	Female	Doctor of Nursing Practice	Lecturer	12	Education	collaboration and workflow, Benefits and concerns, Ethical and legal considerations
Participant 19	41	Male	Master of Science in Nursing	Charge nurse	14	Emergency department	Human-tech balance, AI implementation barriers
Participant 20	30	Female	Master of Science in Nursing	Nurse manager	7	Oncology	Future vision and adaptation, Human-tech balance
Participant 21	49	Female	Masters in Public Health	Senior staff nurse	19	Emergency department	Perceptions of AI readiness, Patient-nurse relationships
Participant 22	36	Male	Masters in Public Health	Senior staff nurse	10	Geriatrics	Future vision and adaptation
Participant 23	28	Female	Masters in Public Health	Lecturer	6	Education	Benefits and concerns, Perceptions of AI readiness

### 2.4 Participant selection

The meticulous process of participant selection encompassed engaging nursing professionals from a broad spectrum of backgrounds, ensuring inclusivity and diversity. Among the essential criteria for inclusion, prospective participants were mandated to hold a minimum of a master's degree and boast a substantial professional history of at least 5 years. In addition, to enrich the study's multidimensional perspective, a specific emphasis was placed on recruiting registered nurses and nurse educators. These individuals were sought not only for their roles within the healthcare landscape but also due to their existing familiarity with the intricate realm of AI concepts within nursing.

Crucially, a pivotal facet of participant selection was the discernible willingness of these professionals to actively partake in insightful conversations [30]. Their preparedness to share nuanced insights and engage in meaningful discussions surrounding the integration of AI in nursing care was a vital determinant in assembling a diverse and dedicated cohort of participants. In combination, these measures ensured that the study's participant selection process was comprehensive, representative, and conducive to a rich exploration of perspectives on the subject matter.

### 2.5 Data collection procedure

The data collection procedure for this study was designed to capture nuanced insights and perspectives from nursing professionals regarding the integration of AI in future nursing care. Firstly, the initial phase involved the identification and selection of potential participants from a diverse pool of nursing professionals. Through targeted outreach within professional networks, healthcare institutions, and nursing organizations, invitations were extended via email and direct communication, inviting individuals to participate in the study. Secondly, prior to the commencement of data collection activities, participants were provided with comprehensive and transparent information detailing the research's objectives, methodologies, potential implications, and ethical considerations [31]. This information was conveyed to ensure participants' full comprehension of their roles and the study's scope. Obtaining informed consent was a paramount step, as participants willingly confirmed their willingness to engage in the study after being informed about the research process, confidentiality measures, and their rights as contributors [32].

In addition to that, data was collected through semi-structured interviews, providing participants with the opportunity to freely express their thoughts on the research topic. The utilization of semi-structured interviews offered flexibility while ensuring comprehensive coverage of relevant areas of inquiry [33]. An interview guide was developed to maintain consistency across interviews. This guide encompassed a series of open-ended questions (Table 2) strategically designed to delve into participants' perspectives concerning the integration of AI in future nursing care. The interviews were thoughtfully conducted in private and comfortable settings, accommodating participants' preferences, and were facilitated either through face-to-face interactions. Thirdly, each interview session was carefully planned to facilitate comprehensive and insightful discussions [34]. Participants were allotted approximately 45–60 min for their interviews, allowing ample time for them to share their thoughts, engage in thoughtful conversations, and delve into the subject matter at a meaningful depth. Finally, to ensure accuracy and fidelity, all interview sessions were audio-recorded with participants' consent. These recordings served as essential resources for subsequent transcription and analysis phases.

**Table 2:** Open-ended questions.

I. How would you describe your understanding of artificial intelligence (AI) in the context of nursing care, and how do you envision its role in shaping the future of healthcare?
II. Can you share any personal experiences or instances where you have encountered or interacted with AI technologies in your nursing practice? How did these experiences influence your perspective on AI's potential impact?
III. In your opinion, what are the potential advantages and challenges associated with integrating AI into nursing care practices? How might these aspects affect patient outcomes and the nursing profession as a whole?
IV. How do you perceive the collaboration between AI technologies and human nurses? What roles and responsibilities do you envision for AI in enhancing patient care, and how do you see these roles evolving over time?
V. What ethical considerations do you believe should be taken into account when integrating AI into nursing care? How can these considerations be effectively addressed to ensure patient safety, privacy, and overall well-being?
VI. How might AI-driven advancements impact the skill sets and competencies required of future nursing professionals? From your perspective, how can nurses best prepare to adapt to these changes and leverage AI technologies to deliver optimal patient care?
VII. Reflecting on your experiences and insights, what recommendations or strategies would you propose to healthcare organizations and nursing education programs to effectively incorporate AI education and training, and ensure a seamless transition to a technologically enhanced nursing landscape?

### 2.6 Data analysis

**Fig. 1:**
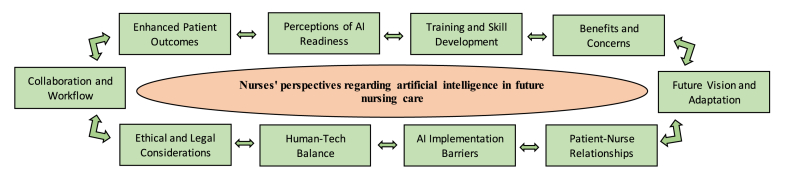
Thematic analysis.

In the process of data analysis, a thematic analysis (Fig. 1) approach was employed to examine the qualitative data garnered from the conducted interviews. This systematic and adaptable methodology offered a comprehensive framework for identifying, interpreting, and elucidating patterns, themes, and insights embedded within the collected data [35]. The analysis process encompassed a series of intricately woven steps, each contributing to a robust and meaningful interpretation of participants' perspectives: Initially, the recorded interviews were transcribed verbatim to ensure the precision of content representation. The transcripts then underwent an extensive phase of data familiarization, wherein the researcher delved into a thorough review and immersion to develop an in-depth comprehension of the narrative richness [36].

Following data familiarization, the researcher embarked upon the task of initial coding, a process that entailed the extraction of fundamental codes signifying significant units of data. These codes were meticulously derived line by line, capturing the nuanced expressions and experiences articulated by the participants. Subsequently, these codes were meticulously organized and amalgamated into potential themes. This process of code collation was characterized by a constant comparative analysis, allowing for the identification of overarching trends and patterns that emerged from the data [37]. As the themes gradually crystallized, they underwent a phase of refinement and development through the grouping of related codes [38]. The researchers scrupulously scrutinized the intricate interplay between themes and codes, ensuring an accurate and authentic portrayal of participants' viewpoints.

### 2.7 Ethical considerations, trustworthiness and rigor

Ethical considerations were rigorously addressed throughout the study. Informed consent was obtained from all participants, ensuring their voluntary participation. Participants' confidentiality and anonymity were strictly maintained during data collection, analysis, and reporting. Ethical guidelines and principles outlined by relevant research ethics committees were adhered to at all stages of the study, including approval from the Mahbubur Rahman Memorial Hospital & Nursing Institute (Approval number: MRMHNI/IRB/HR-03/September 11, 2023).

The trustworthiness of the article was strengthened through a series of rigorous measures meticulously crafted to ensure the research's validity and reliability [39]. Initially, the research design, characterized by a descriptive qualitative approach, was inherently well-suited to capture the nuanced viewpoints of nursing professionals concerning the integration of AI in future nursing care. The utilization of qualitative methods facilitated a comprehensive exploration of participants' perspectives, thereby enhancing the authenticity of the findings. Moreover, the sampling strategy, entailing the purposive selection of a diverse cohort of nursing professionals, contributed significantly to the study's credibility. The inclusion of participants spanning a spectrum of experience and expertise bolstered the depth and breadth of insights, culminating in a well-rounded comprehension of the subject matter. The scrupulous process of data collection, marked by semi-structured interviews and comprehensive interview guides, further fortified the study's credibility. These methodological choices not only encouraged candid participant responses but also minimized potential biases, ensuring a heightened accuracy in the shared information. Furthermore, the study's commitment to trustworthiness was further accentuated by the rigorous approach to data analysis, primarily through thematic analysis. This methodical journey encompassed coding, theme development, and member checking, collectively enhancing the dependability and authenticity of the identified patterns and themes.

## 3 Results

### 3.1 Perceptions of AI readiness

This study revealed that nurses' viewpoints on AI readiness encompass a blend of optimism and caution. They see the integration of AI as an opportunity to enhance patient care and streamline processes, recognizing its potential to redefine healthcare. This optimism, however, is paralleled by concerns about the preservation of their expertise and the unique human connection central to nursing. Their readiness to embrace AI is coupled with a call for comprehensive training, highlighting their commitment to ensuring a seamless integration that leverages AI's benefits [40]. The nurses' adaptability shines through, as they acknowledge AI's alignment with their innovative spirit and express their willingness to evolve with the technology. While acknowledging initial apprehensions, the nurses' unwavering belief in education and training underscores their confidence in AI integration. They envision AI as a tool to amplify their caregiving role rather than replace it, emphasizing the holistic approach that defines nursing.

"As a nurse, I see a momentous opportunity before us – to fully embrace the incorporation of artificial intelligence into nursing care. This step forward holds the promise of elevating patient outcomes and streamlining our care processes through the power of technology." *(Participant 1, female, Senior staff nurse)*

"While the potential of AI in nursing sparks my curiosity, there's a shared concern among my colleagues. We're eager to explore AI's capabilities, yet we're also mindful of preserving the intrinsic value of our expertise and the irreplaceable human connection we offer in nursing." *(Participant 4, female, lecturer)*

"The prospect of integrating AI into nursing is captivating, but it comes with a responsibility to ensure proper training. Our nursing community is genuinely open to this progression, provided we're equipped with the skills needed to harness AI's potential effectively." *(Participant 11, male, charge nurse)*

"Nurses are renowned for our adaptability, and the integration of AI aligns with our spirit of innovation. We recognize that AI could redefine patient care, and I, along with many colleagues, am ready to embrace this technological evolution to advance our field." *(Participant 23, male, lecturer)*

"While the concept of AI might be met with apprehension, I firmly believe that through comprehensive education and training, we can not only welcome AI but also adeptly integrate it into nursing practice, enhancing our role as skilled caregivers." *(Participant 21, female, Senior staff nurse)*

### 3.2 Benefits and concerns

In this study, nurses' discourse on the benefits and concerns surrounding AI in healthcare illustrates a delicate interplay between optimism and caution. The prospect of AI's contribution to early detection of critical medical indicators sparks excitement, offering the potential to enhance swift interventions and ultimately save lives [41]. However, a notable apprehension looms – the possibility that AI might overlook subtle nuances perceivable only by a seasoned human observer [42]. The thought of AI relieving nurses from routine tasks is invigorating, envisioning a future where professionals can channel their energies towards intricate patient needs and fostering deeper connections. This vision aligns with the nurses' commitment to providing holistic care that extends beyond clinical procedures [43].

As AI integration progresses, the nurses' emphasis on safeguarding patient data privacy and security resonates deeply. This cautious approach highlights their dedication to maintaining the trust and confidentiality bestowed upon them by patients. In this evolving landscape, nurses' dedication to their role as caregivers remains steadfast. The potential benefits of AI are acknowledged, yet the nurses' unwavering commitment to compassionate care remains the cornerstone. Their quotes reflect a nuanced interplay between embracing innovation and ensuring that the essence of nursing – the human touch – is preserved amidst technological advancements.

"The idea of AI aiding in the early detection of critical medical indicators is undeniably exciting, holding the potential to intervene swiftly and save lives. However, there's a prevailing concern that AI might overlook nuanced aspects that only a trained human eye could catch." *(Participant 9, female, lecturer)*

"It's genuinely invigorating to consider how AI could alleviate us from mundane tasks, freeing up nurses to concentrate on intricate patient needs and fostering more profound connections with those we care for." *(Participant 14, female, Senior staff nurse)*

"Given the sensitive nature of patient data, a cautious approach is essential in implementing AI. We must prioritize safeguarding patient privacy and data security as we navigate the integration of this technology." *(Participant 18, female, lecturer)*

"Our patients entrust us with their well-being, relying on our expertise and compassionate touch. The potential of AI to support various healthcare aspects is undeniable, but it's crucial that we proceed thoughtfully to maintain the dedicated care that defines us." *(Participant 23, male, lecturer)*

### 3.3 Enhanced patient outcomes

Nurses' quotes delve into the realm of enhanced patient outcomes through AI integration, evoking a blend of anticipation and hope. The notion of AI foreseeing potential patient deteriorations before they manifest sparks excitement, hinting at the possibility of timelier interventions and improved clinical results [44]. In addition, imagining treatment plans meticulously tailored to individual patient needs, guided by AI's analytical prowess, opens the door to a new era of patient care. This concept promises a level of personalization previously unattainable, with the potential to redefine the very essence of healthcare [45].

Moreover, the rapid analytical capabilities of AI offer the prospect of swifter insights and more immediate decision-making in patient treatment, particularly critical in life-or-death situations. Here, time emerges as a pivotal factor, where AI's efficiency could be a game-changer. Furthermore, as nurses, the central focus remains unwavering – the well-being of patients. The nurses' willingness to explore AI's potential contribution to diagnoses and customized treatment strategies speaks to their dedication. This avenue of exploration aligns with their commitment to providing the best care possible, suggesting a future where AI augments their capabilities in service of patients' welfare.

"The mere idea of AI predicting potential patient deteriorations before they even surface ignites a blend of excitement and optimism. This innovation could potentially translate into more prompt interventions and ultimately result in enhanced clinical outcomes." *(Participant 6, female, nurse manager)*

"Picture this: treatment plans meticulously crafted to suit the distinct needs of each patient, guided by the analytical prowess of AI. This concept holds the potential to redefine patient care, tailoring it to a degree we've never experienced before." *(Participant 10, female, clinical instructor)*

"I am sure that the rapid pace at which AI can analyze and interpret medical data offers the promise of quicker insights and more immediate decision-making in patient treatment. Mmmmm …. In critical scenarios, time truly becomes a critical factor that can mean the difference between life and death." *(Participant 7, female, Senior staff nurse)*

"At our core, as nurses, our paramount goal is the well-being of our patients. If AI has the potential to contribute to this noble cause by enhancing diagnoses and tailoring treatment strategies, then it's an avenue we must wholeheartedly explore and integrate." *(Participant 9, female, lecturer)*

### 3.4 Collaboration and workflow

This study found that nurses' quotes illuminate the significance of collaboration and workflow enhancement through AI integration. Nursing thrives on teamwork, and the prospect of AI as a seamless partner, streamlining tasks and fostering coordination, presents an enticing avenue for improved efficiency. Moreover, AI's potential to amplify the collective expertise of healthcare teams resonates strongly. Envisioning AI as an integral addition, providing valuable insights for shared decision-making, hints at a future where technology seamlessly complements human expertise [46]. In addition to that, the nurses' vision underscores the pivotal role of effective communication among healthcare professionals. AI's facilitative role in information sharing aligns with their commitment to optimizing patient outcomes, demonstrating a forward-looking approach where collaboration and technology intertwine for the betterment of patient care.

"Nursing is all about teamwork and collaboration. AI, when seamlessly integrated, could become a valuable partner in our efforts, streamlining tasks and enhancing coordination among healthcare professionals." *(Participant 5, male, charge nurse)*

"Our healthcare team's strength lies in the collective expertise we bring. If AI is effectively harnessed, it could amplify our collaborative spirit, leading to a more comprehensive and well-rounded approach to patient care." *(Participant 1, female, Senior staff nurse)*

"When I think about the future, I envision AI as an indispensable addition to our team, offering us insights and data-driven perspectives that contribute to our shared decision-making process." *(Participant 18, female, lecturer)*

"Effective communication among healthcare professionals is paramount. AI should play a facilitating role, helping us share critical information and insights to ultimately enhance patient outcomes." *(Participant 15, female, charge nurse)*

### 3.5 Human-tech balance

In this research, nurses' discourse eloquently addresses the delicate balance between humanity and technology in nursing care. As AI integration advances, the nurses emphasize the enduring essence of their role – one that extends beyond mere technology. Patients seek not just clinical expertise, but also the warmth of compassion and understanding, virtues that transcend AI's capabilities. In addition, nurses believed that nursing is depicted as a tapestry interwoven with empathy and genuine human connections. While AI contributes technical support, it's the nurses' unique ability to forge authentic bonds that distinguishes their practice [47].

Furthermore, nurses mentioned that the envisioned partnership between nurses and AI is portrayed as symbiotic, with AI handling analytical tasks while nurses focus on the human facets of patient care. This synergy enhances their practice, allowing them to deliver the compassionate touch patients seek. Despite AI's potential for medical progress, the nurses' unwavering commitment to compassionate care stands as the bedrock of nursing. Their statements underscore a collective belief in AI's potential to augment nursing, provided it complements and strengthens the deeply rooted values that define this noble profession.

"As we explore the integration of AI into nursing care, it's crucial to remember that our role extends far beyond technology. Patients seek not only medical expertise but also compassion and understanding – qualities that go beyond what AI can provide." *(Participant 17, female, Senior staff nurse)*

"Nursing is a rich tapestry woven with empathy, compassion, and genuine human connections. While AI can certainly offer technical support, it's our unique ability to form real connections with patients that truly sets us apart." *(Participant 19, male, charge nurse)*

"The partnership between nurses and AI should be symbiotic, enhancing our practice by allowing us to focus on the deeply human aspects of patient care while AI handles the more analytical tasks." *(Participant 4, female, lecturer)*

"While AI may lead us forward in terms of medical progress, it's important to approach it with the understanding that our commitment to compassionate care remains the foundation of nursing. AI should reinforce this dedication rather than diminish it." *(Participant 20, female, nurse manager)*

### 3.6 Training and skill development

This study found nurses' important quotes that delves into the imperative of training and skill development in the context of AI integration in nursing care. This evolving landscape demands a thorough reassessment of training programs to ensure nurses possess the aptitude to navigate AI seamlessly within their practice [48]. In addition, the nurses' commitment to embracing AI is underscored by a dedication to continuous learning. They emphasize the significance of actively seeking opportunities to enhance their AI-related understanding, highlighting its pivotal role in delivering exceptional patient care.

This study also explored that nurses believe AI's potential in nursing is acknowledged as a frontier of innovation, necessitating an investment in training that empowers nurses to harness its capabilities effectively. Skill development is presented as a pathway to unlocking AI's potential, positioning nurses at the forefront of patient care through a harmonious blend of clinical expertise and AI utilization. The nurses' readiness to adapt their skillset exemplifies their proactive approach to embracing technological advancements. As the integration of AI gains momentum, nursing education's evolution becomes crucial, engaging both aspiring and seasoned nurses in a journey of learning that ensures harmonious collaboration with AI-driven systems.

"Bringing AI into nursing care necessitates a comprehensive reevaluation of our training programs. Equipping ourselves with the skills to navigate this technology confidently and ensure its seamless integration into our practice is essential." *(Participant 2, male, nurse manager)*

"Embracing AI as nursing professionals requires an ongoing commitment to learning. Actively seeking opportunities to enhance our understanding of AI and its applications is crucial for delivering the highest quality care." *(Participant 12, female, nurse manager)*

"AI presents an exciting frontier for nursing, but effective utilization requires investing in training that empowers us to harness its capabilities. By continuously developing our skills, we can ensure that AI becomes a powerful tool in our caregiving arsenal." *(Participant 16, male, lecturer)*

"The integration of AI calls for a shift in our skillset. Our dedication to acquiring the necessary expertise is key to remaining at the forefront of patient care, effectively complementing our clinical acumen with AI." *(Participant 8, male, Senior staff nurse)*

"To fully unlock the potential of AI, nursing education needs to adapt and evolve. As aspiring and practicing nurses, we should actively engage in learning experiences that prepare us to seamlessly collaborate with AI-driven systems." *(Participant 13, male, lecturer)*

### 3.7 Ethical and legal considerations

This study revealed that nurses undertake a comprehensive exploration of the ethical and legal dimensions linked to the integration of AI into nursing practice. With AI's emergence in their domain, nurses keenly recognize the intricate ethical inquiries it prompts, especially concerning patient privacy and data security. They proactively adopt the role of advocates, embracing the responsibility to champion these concerns and ensure the conscientious and ethical use of AI. Within their perspective, nurses acknowledge the delicate balance between innovation and ethical standards. They accentuate the need for discourse aimed at establishing frameworks that guide the ethical incorporation and application of AI within nursing. This shared responsibility underscores their unwavering commitment to upholding the core values and integrity inherent in their profession [49].

Moreover, nurses emphasized the paramount importance of safeguarding patient rights and confidentiality. Their proactive involvement in discussions surrounding AI's implementation highlights their pivotal role in shaping a future where AI seamlessly aligns with ethical imperatives, preserving the foundational trust that underpins their practice. Ultimately, the nurses' discourse accentuates their pivotal role as guardians of patient care, entrusted with the ethical utilization of AI. Their steadfast commitment to maintaining ethical boundaries exemplifies their dedicated adherence to the fundamental principles and integrity intrinsic to the nursing profession.

"As AI enters our domain, critical ethical questions arise, particularly concerning patient privacy and data security. It falls upon us, as nursing professionals, to champion these considerations and ensure the ethical and responsible use of AI." *(Participant 18, female, lecturer)*

"The use of AI requires a delicate balance between innovation and upholding legal and ethical standards. Engaging in discussions to establish frameworks guiding the ethical implementation and utilization of AI in nursing is our collective responsibility." *(Participant 9, female, lecturer)*

"As we navigate uncharted waters with AI, safeguarding patient rights and privacy remains paramount. Beyond caregiving, we must advocate for the ethical and legal implications of AI, preserving the core values of our profession." *(Participant 3, female, Senior staff nurse)*

"The ethical landscape surrounding AI is intricate. By actively participating in conversations about its implementation, nursing professionals play a crucial role in shaping a future where AI seamlessly integrates, aligning with our ethical obligations." *(Participant 12, female, nurse manager)*

"Nursing professionals stand at the forefront of patient care. We are entrusted with ensuring that AI's potential is harnessed within ethical boundaries, upholding the trust and integrity that define our profession." *(Participant 16, male, lecturer)*

### 3.8 AI implementation barriers

The nurses' dialogue delves into the critical realm of AI implementation barriers in nursing. While the potential of AI is vast, the nurses recognize the need to surmount obstacles such as limited resources and resistance to change for a successful integration. A collaborative approach to address these challenges is underscored as pivotal for a seamless transition. Regarding this, AI's integration demands a united endeavor to overcome technical complexities and entrenched resistance within healthcare systems [50]. By acknowledging and collectively addressing these barriers, the nurses highlight the potential to unlock the transformative capabilities of AI. In addition, nurses believe that success in AI integration hinges upon navigating and triumphing over implementation challenges. Fostering a culture of adaptability and innovation, the nurses advocate for a proactive stance that equips them to collectively surmount barriers and seamlessly incorporate AI.

While embracing AI brings forth challenges, the nurses' unwavering determination propels them forward. By identifying and tackling implementation barriers, they ensure AI's integration as an invaluable tool, ultimately enhancing their capacity to deliver optimal patient care. The nurses' quotes spotlight the necessity for a comprehensive strategy that directly confronts implementation hurdles. Through collaborative efforts and an adaptive mindset, nursing professionals position themselves to conquer obstacles, harness AI's potential benefits, and pave the way for a technologically advanced future in patient care.

"While the potential of AI in nursing is immense, overcoming barriers like limited resources and resistance to change is essential for successful implementation. Addressing these challenges collectively is crucial for a smooth transition." *(Participant 11, male, charge nurse)*

"AI implementation requires a concerted effort to overcome technical hurdles and resistance within our healthcare systems. Recognizing and collaboratively addressing these barriers can unlock the transformative power of AI." *(Participant 8, male, Senior staff nurse)*

"Successfully integrating AI hinges on navigating and surmounting implementation challenges. Cultivating adaptability and fostering innovation as a culture equips us to collectively overcome barriers, allowing AI to be seamlessly incorporated." *(Participant 4, female, lecturer)*

"While embracing AI presents challenges, it's imperative that we move forward. Identifying and addressing implementation barriers ensures that AI becomes an indispensable tool, enhancing our ability to provide optimal patient care." *(Participant 17, female, Senior staff nurse)*

"Integrating AI necessitates a comprehensive strategy to address barriers head-on. Through proactive collaboration and a willingness to adapt, nursing professionals can overcome obstacles and harness AI's potential benefits." *(Participant 19, male, charge nurse)*

### 3.9 Patient-nurse relationships

The nurses' conversation centers on the invaluable patient-nurse relationships within nursing practice. They advocate for a harmonious integration of AI that augments, rather than substitutes, their capacity to connect with and understand the unique needs of patients. AI's role is envisioned as enhancing patient interactions, extending the time available for nurturing profound relationships, and providing the emotional support that patients rely upon [51]. While AI offers data-driven insights, the nurses emphasize that their aptitude for delivering compassionate care remains unmatched [52]. They assert that despite AI's potential to revolutionize patient care, upholding the human touch remains paramount, preserving the essence of nursing and genuine patient connections.

"Our connection with patients forms the cornerstone of nursing practice. AI should complement, not replace, our ability to connect with and comprehend the unique needs of those under our care." *(Participant 13, male, lecturer)*

"AI should enhance our patient interactions, not replace them. I think, by streamlining tasks, AI affords us more time to build meaningful relationships and offer the emotional support patients rely on." *(Participant 10, female, clinical instructor)*

"A meaningful patient-nurse relationship goes beyond technology. While AI offers data-driven insights, our capacity to deliver compassionate care remains unparalleled and central to our role." *(Participant 21, female, Senior staff nurse)*

"Though AI may revolutionize patient care, it's paramount to uphold the human element. AI should complement our ability to genuinely connect with patients, preserving the core essence of nursing." *(Participant 5, male, charge nurse)*

### 3.10 Future vision and adaptation

The nurses' statements revolve around a visionary outlook and adaptive stance towards AI's integration. Envisioning a future where AI synergizes with their capabilities underscores the need for proactive adaptation. They emphasize that staying informed, embracing innovation, and maintaining an open-minded perspective are pivotal in shaping a future where AI elevates their practice. In addition, the nurses regard AI's integration as the herald of a transformative epoch in nursing. Their embrace of change and forward-looking mindset positions them to harness AI's potential, emerging as leaders in an evolving landscape where innovation prevails. Amidst evolving nursing care, adaptation takes precedence. Their vision of a future harmonizing AI underscores their role as drivers of innovation, shaping a path where novel technologies and compassionate patient care seamlessly converge.

The nurses' candid perspective acknowledges the inextricable link between nursing care and AI's trajectory. Their readiness to embrace new technologies and recalibrate their approach positions them to influence a future where cutting-edge innovations enhance their dedication to patient well-being. By proactively anticipating and embracing AI's potential, the nurses carve a trail towards pioneering nursing's future [53]. Their commitment to preparation stands as a testament to their dedication, contributing to a future where the integration of innovative technologies magnifies their mission of effective and empathetic patient care.

"Envisioning a future where AI augments our capabilities demands proactive adaptation. Staying informed, embracing innovation, and maintaining an open-minded perspective shapes a future where AI enhances our practice." *(Participant 15, female, charge nurse)*

"AI's integration heralds a transformative era in nursing. Embracing change and fostering a forward-looking mindset enables us to maximize AI's potential benefits and position ourselves as leaders in this evolving landscape." *(Participant 6, female, nurse manager)*

"Adaptation is essential as nursing care evolves. Envisioning a future where AI seamlessly integrates empowers nursing professionals to drive innovation and lead the way in this paradigm shift." *(Participant 20, female, nurse manager)*

"To be honest, nursing care's future intertwines with AI. I believe, by embracing new technologies, challenging norms, and adapting our approach, we can collectively shape a future where innovative technologies align seamlessly with compassionate patient care." *(Participant 22, male, Senior staff nurse)*

"Anticipating and embracing AI's potential positions us as pioneers in shaping nursing's future. Through proactive preparation, we contribute to a future where innovative technologies enhance our commitment to effective and compassionate patient care." *(Participant 3, female, Senior staff nurse)*

## 4 Discussion

The findings of this research provide a comprehensive understanding of nurses' perceptions regarding the integration of AI into nursing care. The themes that emerged from the participants' narratives shed light on their readiness, expectations, concerns, and aspirations for the future of nursing practice in the era of AI.

Nursing professionals participating in this study conveyed a collective sense of preparedness and optimism when it comes to embracing the integration of AI into nursing care. They acknowledge the potential of AI to amplify patient outcomes and optimize care processes through technological advancements. This shared perspective resonates with prior research, underlining nurses' enthusiasm in exploring the potential contributions of AI to nursing practice [14,54]. However, amidst their openness, participants also expressed a shared concern about upholding the inherent value of nursing expertise and the indispensable human connection that characterizes nursing care. This apprehension aligns with existing literature, which underscores the significance of maintaining the interpersonal dimension of healthcare even as technology continues to evolve [55].

The fervor surrounding AI's capacity to facilitate early detection and intervention, coupled with its potential to relieve nurses from routine tasks, mirrors findings from earlier studies that accentuate AI's role in enhancing clinical decision-making and optimizing workflow efficiency [56,57]. Participants' emphasis on a cautious approach to AI implementation aligns with research that underscores the imperative of addressing concerns surrounding data security and privacy in the context of AI integration [58]. The participants' unwavering commitment to sustaining dedicated and compassionate care amidst AI integration resonates with studies that emphasize the essential nature of preserving the humanistic essence of caregiving [59,60]. Similarly, the participants' enthusiasm for AI's predictive potential in identifying patient deteriorations finds resonance with existing research highlighting AI's ability to analyze intricate datasets and offer timely insights [61]. The concept of tailoring individualized treatment plans through AI guidance corresponds with studies delving into personalized medicine and its anticipated impact on patient well-being [62,63]. Moreover, the recognition of time as a pivotal factor in patient care aligns with the growing recognition of AI's capacity to expedite decision-making, particularly in critical scenarios [64].

In the participants' view, AI is envisioned as a collaborative partner within healthcare teams, reinforcing research that explores AI's role in augmenting healthcare professionals' capabilities and promoting interdisciplinary collaboration [65]. Their emphasis on effective communication resonates with findings that underscore AI's potential to facilitate seamless information exchange and enhance the coordination of care [66]. Regarding this, the participants' accentuation of the symbiotic partnership between nursing professionals and AI, with AI enhancing nursing practice while preserving the unique human touch, echoes research discussing AI as a supportive tool that supplements, rather than supplants, the role of healthcare providers [67]. Their unwavering dedication to delivering compassionate care in the face of technological advancements aligns with studies advocating for the integration of AI in alignment with patient-centered values [68].

Moreover, the participants' acknowledgment of the necessity for comprehensive training and continuous skill enhancement in AI integration is harmonious with research emphasizing the pivotal role of nursing education in effectively harnessing AI technologies [54]. Their proactive stance towards learning and adapting their skillset reflects a heightened awareness of the evolving demands of nursing practice in the era of AI [69]. In terms of this, the participants' unwavering commitment to ethical and responsible AI utilization resonates with findings from studies highlighting the significance of addressing ethical and legal ramifications in the integration of AI [40]. Their advocacy for patient rights and privacy aligns with research underscoring nurses' accountability in ensuring the ethical integration of AI [70]. In addition to that, the participants' acknowledgment of obstacles and the necessity for collective efforts to overcome these challenges aligns with studies that delve into the hurdles of AI implementation and advocate for cultivating an innovative organizational culture [19]. Their resolute determination to harness the potential benefits of AI is in sync with research emphasizing AI's transformative potential within the realm of healthcare [71].

The participants' emphasis on nurturing patient-nurse relationships amidst AI integration corresponds with research that underscores AI's role in enhancing, rather than replacing, these human connections [58]. Their commitment to providing emotional support underscores the paramount importance of nursing professionals in delivering holistic and empathetic care [61,72]. Furthermore, the participants' forward-looking perspective on AI integration resonates with studies discussing AI's transformative potential in shaping the trajectory of nursing practice [41]. Their readiness to embrace novel technologies and challenge traditional norms mirrors research advocating for active nursing involvement in shaping the future of healthcare [73].

## 5 Implications of this study

This study provides substantial implications for the healthcare industry. Firstly, it underscores the growing acceptance of AI in nursing care, indicating a paradigm shift in healthcare delivery. As nursing professionals express positive attitudes toward AI, healthcare institutions should consider integrating AI technologies into their care processes [74]. Moreover, understanding the concerns and expectations of nursing professionals regarding AI is crucial. It highlights the need for targeted training programs to ensure that nurses are well-prepared to work alongside AI systems effectively. Training should not only encompass technical aspects but also ethical considerations to maintain patient-centric care. Additionally, this study highlights the importance of interdisciplinary collaboration. The involvement of AI experts, nurses, and other healthcare stakeholders in the development and implementation of AI tools can lead to more tailored and beneficial solutions. This collaboration can foster innovation in nursing care, ultimately improving patient outcomes.

## 6 Recommendations

Based on the findings of this study, several recommendations can be made. Firstly, healthcare institutions should invest in AI education and training programs for nursing professionals. These programs should cover both the technical aspects of AI and the ethical considerations involved in its use. Continuous learning and upskilling will be crucial as AI technologies evolve. Secondly, interdisciplinary collaboration should be encouraged. AI developers, healthcare administrators, nurses, and patients should work together to design AI systems that are patient-centered, user-friendly, and aligned with healthcare goals. Thirdly, healthcare institutions should adopt a phased approach to AI implementation. Start with simple AI applications, like predictive analytics for patient outcomes, before moving on to more complex tasks. This gradual integration will allow nurses to become familiar with AI and its benefits.

## 7 Strengths and limitations

One strength of this study is its qualitative approach, which allowed for in-depth exploration of nursing professionals' perspectives. The use of interviews and thematic analysis provided rich data for a comprehensive understanding of the subject. However, there are limitations to consider. Firstly, the study's sample size may not represent the diversity of nursing professionals' perspectives. Future research could aim for a larger and more diverse sample to capture a broader range of opinions. Secondly, the study focused on the perspectives of nursing professionals, but it did not include the viewpoints of patients and AI developers. Incorporating these perspectives could provide a more holistic understanding of AI in nursing care.

## 8 Conclusions

This study concludes that AI is not a threat to nursing but rather an opportunity for growth and enhanced care delivery. The future of nursing care is a dynamic amalgamation of human expertise and AI, a synergy that has the potential to redefine healthcare. The key lies in nurturing this partnership with the right knowledge, collaboration, and ethical principles, ensuring that the ultimate beneficiaries are the patients who receive more effective, efficient, and compassionate care. As AI technologies continue to advance, nursing care stands on the brink of a transformative era. With careful planning, education, and collaboration, AI can become a valuable tool in the hands of nursing professionals, ultimately improving healthcare delivery and patient outcomes.

## CRediT authorship contribution statement

**Moustaq Karim Khan Rony:** Writing – review & editing, Writing – original draft, Visualization, Validation, Supervision, Software, Resources, Project administration, Methodology, Investigation, Formal analysis, Data curation, Conceptualization. **Ibne Kayesh:** Writing – review & editing, Writing – original draft, Validation, Investigation, Formal analysis, Data curation, Conceptualization. **Shuvashish Das Bala:** Writing – review & editing, Supervision, Software, Resources, Investigation, Formal analysis, Data curation. **Fazila Akter:** Writing – original draft, Visualization, Project administration, Investigation, Formal analysis, Data curation. **Mst Rina Parvin:** Writing – review & editing, Writing – original draft, Visualization, Validation, Supervision, Software, Project administration, Methodology, Formal analysis, Data curation, Conceptualization.

## Declaration of competing interest

The authors declare that they have no known competing financial interests or personal relationships that could have appeared to influence the work reported in this paper.
